# Mutations in the plasma cell clone identify mechanism of polyneuropathy in a case of POEMS syndrome associated with Castleman disease and multiple myeloma

**DOI:** 10.1007/s00277-022-05032-1

**Published:** 2022-11-19

**Authors:** Annamaria Brioli, Antje Wyrwa, Ulrike Rüddel, Olaposi Yomade, Udo Lindig, Wiebke Franz, Hans-Heinrich Wacker, Nikolaus Gaßler, Stefan Schweyer, Ha-Yeun Chung, Hubertus Axer, Otto W. Witte, Andreas Hochhaus, Karin G. Schrenk

**Affiliations:** 1 Abteilung Hämatologie und Internistische Onkologie, Klinik für Innere Medizin II, Universitätsklinikum Jena, Am Klinikum 1, Jena, 07747 Germany; 2 Klinik und Poliklinik für Innere Medizin C, Hämatologie und Onkologie, Universitätsmedizin Greifswald, Greifswald, Germany; 3 Hämatopathologie Schleswig-Holstein, Kiel, Germany; 4 Institut Für Rechtsmedizin, Sektion Pathologie, Universitätsklinikum Jena, Jena, Germany; 5 Pathologie Starnberg, Starnberg, Germany; 6 Klinik Für Neurologie, Universitätsklinikum Jena, Jena, Germany

Dear Editor,

POEMS (polyneuropathy, organomegaly, endocrinopathy, M-protein, and skin changes) is a paraneoplastic syndrome associated with plasma cell neoplasia. The diagnosis of POEMS syndrome requires the two mandatory criteria polyneuropathy and monoclonal plasma cell proliferation as well as one of the three other major criteria (Castleman disease [CD], sclerotic bone lesion, and elevated vascular endothelial growth factor [VEGF]) and one of the six minor criteria [1]. Castleman disease is a rare heterogeneous group of lymphoproliferative disorders. According to the 5th edition of the WHO classification of hematolymphoid tumors, CD is now classified as tumor-like lesion with B-cell predominance [2]. Based on the distribution of the lymphadenopathy localized, unicentric CD (UCD) is distinguished from multicentric CD (MCD). Histological variants include the hyaline vascular type with aggregates of follicular dendritic cells and surrounding lymphocytes forming “onion skin” layers around the dendritic cells, the plasma cell variant, and the mixed subtype. MCD may be associated with human herpesvirus-8 (HHV-8) or human immuno-deficiency virus (HIV) infections. Increased interleukin-6 (IL-6) expression has been shown to be associated with CD and promotes the secretion of VEGF [3]. In CD without clonal plasma cell disorder, the Castleman disease variant of POEMS syndrome may occur and usually presents as a less severe and sensory polyneuropathy [4]. Due to its rarity, POEMS syndrome is very difficult to diagnose, and patients often present with severe polyneuropathy. The mechanisms by which the small plasma cell clone or the lymphoproliferative clone of the Castleman disease variant of POEMS syndrome cause polyneuropathy are still poorly understood. Here we present the case of a patient with simultaneous CD, solitary plasmacytoma with minimal bone marrow involvement, and POEMS syndrome followed by progression into multiple myeloma (MM). Molecular analysis of both the lymph node and of the bone marrow identified mutations in genes associated with neurodegeneration in the main malignant clone.

**Fig. 1:**
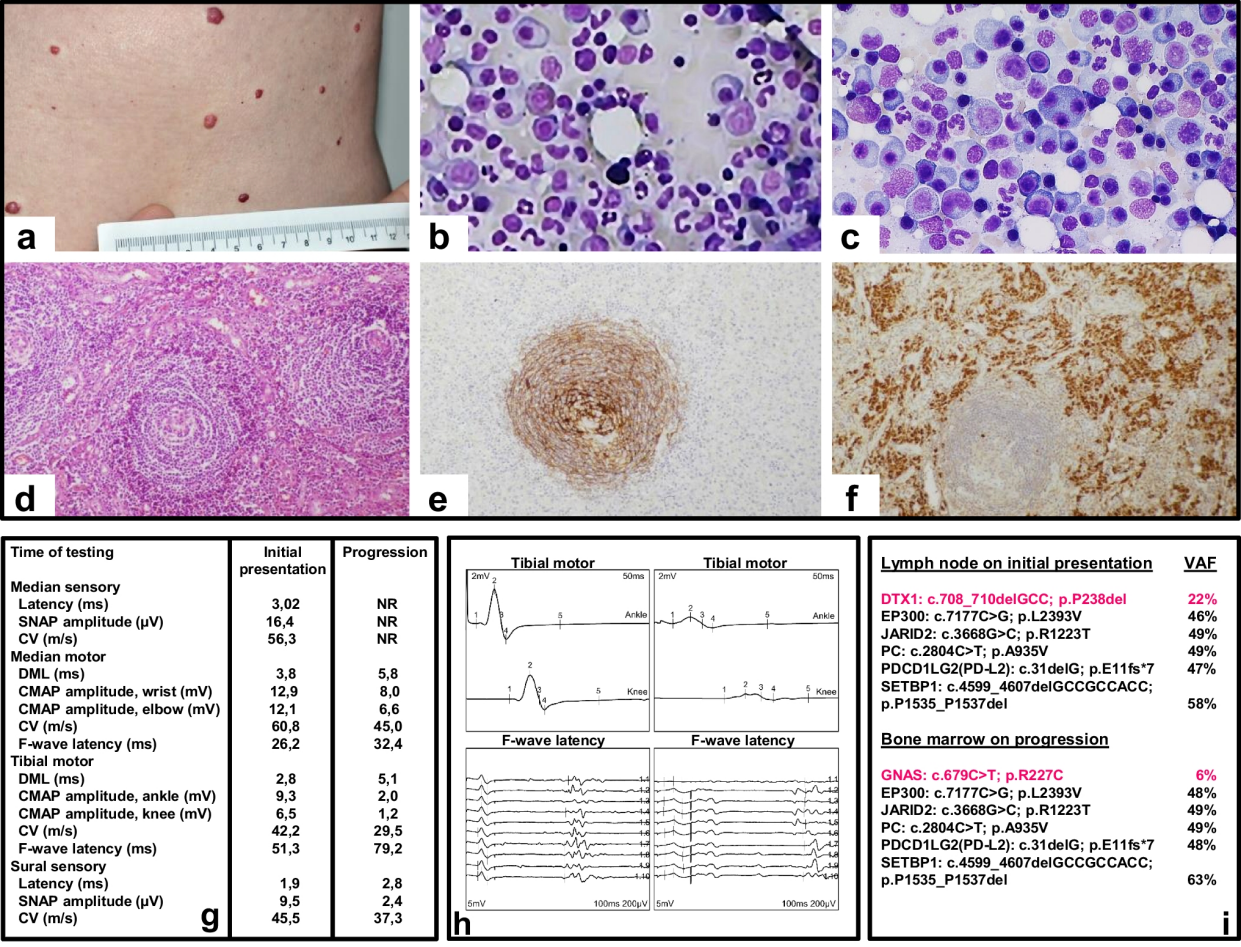
*Upper and middle pane*l: Multiple hemangiomas of the skin **a**, bone marrow biopsy at initial presentation revealed polyclonal plasma cells (400 × magnification) **b**, with progression to multiple myeloma (400 × magnification) **c**. Hematoxylin–eosin staining of lymph node with onion skin layers (10 × magnification) **d**, staining of dendritic reticulum cells (KiM4P 10 × magnification) **e,** and of lambda light chain (10 × magnification) **f**. *Lower panel*: Electrophysiological studies with no pathological findings on initial presentation **g-h**, decrease in conduction velocity and elongation of F-wave latencies after progression into multiple myeloma **g**-**h**. NR, no response. NGS-analysis of initial lymph node biopsy and bone marrow after progression into multiple myeloma demonstrating differential alteration of DTX1 in lymph node biopsy and GNAS in bone marrow biopsy on progression into multiple myeloma. EP300, JARID2, PC, PD-L2, and SETBP1 alterations were found in both biopsies. VAF, variant allele frequency **i**

A 67-year-old woman presented with weight loss, generalized neuropathic pain, disseminated hemangiomas of the skin (Fig. 1a), and enlarged mediastinal lymph nodes. Histology revealed the typical morphology of the hyaline vascular type of CD with hyperplastic lymphoid follicles in combination with a plasmacytoma. By multiplex PCR of the immunoglobulin heavy chain and light chain gene, monoclonal rearrangement was demonstrated, identifying CD and plasmacytoma as two distinct histological entities. Plasma cell variant of CD was excluded (Fig. 1d–f). In bone marrow biopsy, only polyclonal plasma cells and no cytogenetic aberrations were found (Fig. 1b). Despite symptoms of sensory polyneuropathy, electrophysiological studies did not demonstrate any abnormalities at initial presentation (Fig. 1g–h). VEGF serum levels were increased with > 1000 pg/ml (normal range < 445 pg/ml). HIV and HHV-8 were not detectable. There was no evidence of amyloidosis. Because of the multicentric presentation of CD, systemic therapy with one course of prednisone, vincristine, and cyclophosphamide followed by the anti-interleukin-6 antibody siltuximab was commenced. After 13 months of treatment, VEGF levels had decreased to 300 pg/ml; however, the patient experienced multiple cerebral thromboembolic events with ischemic lesions and thrombosis of the left sigmoid sinus. Treatment was changed to immunochemotherapy with 6 courses of R-CHOP (rituximab, cyclophosphamide, doxorubicin, vincristine, prednisone). During the 6th treatment course, lambda light chains increased noticeably. Restaging with lymphadenectomy proved complete remission of CD and persistence of plasmacytoma. Bone marrow biopsy showed progression into multiple myeloma IgA lambda with an increase of plasma cells up to 39% on average (Fig. 1c). Electrophysiological studies revealed a decrease in nerve conduction velocity and elongation of F-wave latencies (Fig. 1g–h). The initial intention to irradiate the mediastinal plasmacytoma was changed to systemic therapy with daratumumab, melphalan, and prednisone (Dara-MP). Due to the progressive peripheral neuropathy, bortezomib was not applied. The patient experienced an allergic reaction to daratumumab according to common toxicity criteria (CTC) grade 3, and therapy was continued with MP followed by lenalidomide and irradiation of osteolytic lesions on progression. Molecular analysis using next-generation sequencing (NGS, FoundationOne Heme, Penzberg, Germany) of the lymph node biopsy at initial presentation and of the bone marrow on progression into multiple myeloma revealed several genetic alterations. Mutation in Deltex (DTX1) was exclusively found in lymph node, whereas mutation in guanine nucleotide-binding protein subunit alpha (GNAS) was only detected in the bone marrow. Identical alterations of EP300, JARID2, PC, PD-L2, and SETBP1 were identified in both biopsies (Fig. 1i).

This case report demonstrates the variable association between Castleman disease and plasma cell dyscrasias as a dynamic process. At initial presentation, Castleman disease and plasmacytoma could be identified as two distinct disease entities in lymph node biopsy, and the patient presented with symptoms of sensory neuropathy with no changes on electrophysiological exam. At the time of progression of the monoclonal plasma cell disorder, electrophysiological exam demonstrated sensory and motor polyneuropathy with a decrease in motor conduction velocity and elongation of F-wave latencies. Reviewing retrospectively the evolution of the case, it is likely that the POEMS syndrome was associated to the minimal systemic clonal plasma cell population from disease onset seen at the time of presentation of the solitary plasmacytoma with minimal bone marrow involvement. Whether a more plasma cell–directed therapy at initial presentation would have modified the course of the neurologic disorder can only be speculated on. It has to be noted however that, at the time of initial presentation, due to the systemic symptoms such as weight loss and the significantly enlarged mediastinal lymph nodes, CD was the disease requiring immediate attention and treatment.

Several molecular abnormalities have been described in CD, multiple myeloma, and POEMS syndrome. Butzmann et al. [5] identified molecular abnormalities in UCD predominantly in mitogen-activating protein kinase (MAPK) and interleukin signaling pathways, whereas in MCD, mostly genes affecting chromatin organization and methylation were mutated [5]. MM is more complex, with molecular abnormalities present at the genetic as well as at the epigenetic level. Interestingly, the molecular analysis in our patient showed the presence of clonal evolution starting from a common progenitor. Five of the seven mutations identified were present both in the lymph node and in the bone marrow, suggesting a common founding clone, followed by branching evolution. This hypothesis is supported by the variant allele frequency (VAF) of the different mutations (Fig. 1i), indicating the presence of EP300, JARID2, PC, PD-L2, and SETBP1 at a clonal level and of GNAS and DTX1 at a subclonal level. Two of the five mutated genes are involved in epigenetic regulation (EP300 and JARID2), strengthening the importance of epigenetic changes in the development of B-cell malignancies. EP300 has been found to be mutated both in MM [6] and in POEMS syndrome [5], while JARID2, although not formally identified as a recurrent mutated gene in MM, locates next to known susceptibility loci for MM and acts as a cofactor of the epigenetic regulator polycomb repressive complex 2 (PRC2). PRC2 has a major impact in the oncogenic transformation and progression of MM, mainly via overexpression and modulation of EZH2 [7]. Two additional mutations in the founding clone (PC and SETPB1) can be linked to neurodegeneration, thus providing a rational for the development of neuropathy in our patient. PC is involved in the synthesis of the neurotransmitter glutamate, and it is intriguing to speculate that alteration in the synthesis of glutamate might have led to or contributed to the peripheral neuropathy, while SETPB1 causes neurodegeneration in Schinzel-Giedion syndrome via p53 inhibition [8]. Different groups have reported that genetic alterations in plasma cells can be linked to the development of neuropathy in myeloma patients, showing a deregulation of genes involved in neurogenesis in the plasma cells of patients developing peripheral neuropathy [9]. The exact mechanism by which alterations in the plasma cells can cause neurodegeneration is still unclear. The ubiquitin ligase DTX1 regulates the Notch pathway which is involved in major regulatory pathways including differentiation and lymphocyte lineage commitment [10]. Interestingly, DTX1 mutation developed only in the lymph node, and mutations of this gene have been identified in patients with diffuse large B-cell lymphoma or follicular lymphoma. GNAS activates protein kinase A (PKA) signaling increasing proliferation and has been reported to be amplified in several cancers. GNAS mutations have been detected in newly diagnosed MM. Whether the occurrence of GNAS mutation was responsible of MM progression remains speculative.

In conclusion we identify for the first time the presence of mutations associated with neurological toxicity and neurodegeneration in the malignant clone of a patient with CD, MM, and POEMS. Whether these features are common to POEMS patients and might contribute to the development of neuropathy needs to be addressed in appropriately designed sequencing studies with a higher number of patients.
